# Functional Characterization of the Ryanodine Receptor Gene in *Diaphorina citri*

**DOI:** 10.3390/life12122005

**Published:** 2022-12-01

**Authors:** Tian-Sheng Liu, Xue-Li Sun, Min-Liang Bin, Gan-Jun Yi, Xin-Xin Zhang

**Affiliations:** 1 Key Laboratory of South Subtropical Fruit Biology and Genetic Resource Utilization (MOA)/Guangdong Province Key Laboratory of Tropical and Subtropical Fruit Tree Research, Institute of Fruit Tree Research, Guangdong Academy of Agricultural Sciences, Guangzhou 510640, China; 2 State Key Laboratory for Conservation and Utilization of Subtropical Agro-Bioresources, College of Life Sciences, South China Agricultural University, Guangzhou 510642, China

**Keywords:** ryanodine receptors, *Diaphorina citri*, mRNA expression, alternative splicing

## Abstract

The Asian citrus psyllid *Diaphorina citri* (Hemiptera: Liviidae) is a major citrus pest spread around the world. It is also a vector of the bacterium ‘*Candidatus* Liberibacter asiaticus’, considered the cause of the fatal citrus disease huanglongbing (HLB). Insect ryanodine receptors (RyRs) are the primary target sites of diamide insecticides. In this study, full-length RyR cDNA from *D. citri* (named DcRyR) was isolated and identified. The 15,393 bp long open reading frame of DcRyR encoded a 5130 amino acid protein with a calculated molecular weight of 580,830 kDa. This protein had a high sequence identity (76–79%) with other insect homologs and a low sequence identity (43–46%) with mammals. An MIR domain, two RIH domains, three SPRY domains, four RyR repeat domains, an RIH-associated domain at the N-terminus, two consensus calcium-binding EF-hands, and six transmembrane domains were among the characteristics that DcRyR shared with insect and vertebrate RyRs. In expression analysis, the DcRyR gene displayed transcript abundance in all tissues and developmental stages as well as gene-differential and stage-specific patterns. In addition, diagnostic PCR experiments revealed that DcRyR had three potential alternative splice variants and that splicing events might have contributed to the various functions of DcRyR. However, diamide resistance-related amino acid residue mutations I4790M/K and G4946E were not found in DcRyR. These results can serve as the basis for further investigation into the target-based diamide pesticide resistance of *D. citri*.

## 1. Introduction

Diamide insecticides are one of the most promising insecticide classes in the twenty-first century, ranking third in the insecticide market in 2018 [1,2]. These insecticides selectively bind and activate the ryanodine receptor (RyR), disrupting calcium (Ca) homeostasis in cells and ultimately killing pests. RyR is a ligand-gated Ca^2+^ channel found on the membranes of muscle cell sarcoplasmic reticulum and nerve cell endoplasmic reticulum [3,4]. Diamide insecticides can be used on various crops because they combine high efficacy against a wide range of pests with an excellent safety profile for mammals [5]. Therefore, these insecticides are ideal for pest control.

The Asian citrus psyllid *Diaphorina citri* Kuwayama (Hemiptera: Liviidae) is a serious pest of Rutaceae, mainly citrus. It damages the citrus plant both directly by feeding and indirectly by vectoring ‘*Candidatus* Liberibacter asiaticus’ (CLas), the bacterial species that causes the highly destructive Asian huanglongbing (HLB; citrus greening disease) [6]. This disease presents a significant threat to citrus production globally [7,8,9,10,11]. HLB has been confirmed in 51 of the 140 citrus-producing countries, causing unprecedented economic losses worldwide [12,13,14]. However, there is no definitive treatment and known resistance for HLB; therefore, controlling pathogenic transmission by *D. citri* is a key approach to preventing the occurrence and spread of HLB [15,16]. The most effective method and primary strategy of managing *D. citri* is to control populations with insecticides [17,18]; however, besides insecticide resistance development, this strategy is unsustainable (i.e., causes environmental pollution) and costly [19,20,21]. Moreover, cases of high-level resistance of *D. citri* to several insecticides have been reported in China, America, Mexico, Brazil, India, and Pakistan, such as neonicotinoids (e.g., imidacloprid and thiamethoxam), organophosphates (e.g., chlorpyrifos and malathion), carbamates (e.g., carbaryl and carbofuran), and pyrethroids (e.g., bifenthrin and fenpropathrin) [19,22,23,24,25,26,27,28,29,30,31]. To date, there are no reports on diamide resistance development in *D. citri*. Nevertheless, with the increase in the application of these chemicals to control *D. citri*, insecticide resistance may occur in the future. Thus, characterizing *D. citri* RyR (DcRyR) is a crucial step to understand the mechanisms underlying insecticide resistance on targeting RyR in *D. citri*.

In this study, full-length DcRyR cDNA was isolated and identified. Furthermore, the gene expression pattern and developmentally regulated alternative splicing of DcRyR were investigated. The findings can provide the basis for the functional characterization of DcRyR and contribute to the discovery of chemicals targeting RyRs and the management of insecticide resistance in *D. citri*.

## 2. Materials and Methods

### 2.1. Insects

A susceptible laboratory population of *D. citri* was reared on *Murraya paniculata* in a greenhouse maintained at 60 ± 5% relative humidity and 27 ± 1 °C with a 16L:8D at the Institute of Fruit Tree Research, Guangdong Academy of Agricultural Sciences. For the detailed information of collected samples in the field see Table S1.

### 2.2. Full-Length Cloning of DcRyR cDNA

Total RNA was extracted from *D. citri* using the GeneJET RNA Kit (Thermo Fisher Scientific, Waltham, MA, USA) according to the manufacturer’s instructions. First-strand cDNA was synthesized using the RevertAid RT Kit (Thermo Fisher Scientific). Seven pairs of special polymerase chain reaction (PCR) primers (S1–S7) were designed according to the alignment between the partial sequence of RyR from *D. citri* (Accession number: XM_017444878.2) and other species (Table S2). Amplification of each fragment was performed in a 50 μL reaction system containing 4 μL of cDNA, 2 μL of each specific primer (10 μM), 25 μL of 2× Phanta Max buffer, 1 μL of dNTP Mix (10 mM each), and 1 μL of Phanta Max Super-Fidelity DNA Polymerase (1 U/μL) (Vazyme, Nanjing, China). The touchdown thermal cycling profile for PCR was initial denaturation at 95 °C for 3 min, 15 cycles at 95 °C for 15 s, 70–55 °C for 20 s (minus 1 °C/cycle), and 72 °C for 90 s; 30 cycles at 95 °C for 15 s, 55 °C for 20 s, and 72 °C for 90 s; and final extension at 72 °C for 5 min. After 2% agarose gel electrophoresis, the PCR products were purified using a Gel Extraction Kit (Omega, Norcross, GA, USA) and inserted into pJET1.2/blunt Cloning Vector (Thermo Fisher Scientific, Waltham, MA, USA). Positive clone samples were extracted and verified by sequencing (Sangon Biotech, Shanghai, China). The complete cDNA of DcRyR was assembled by overlapping all amplified fragments.

### 2.3. Sequencing and Phylogenetic Analysis

The cDNA sequence assembly was performed with the Cap3 program (https://www.ebi.ac.uk/Tools/msa/clustalo/) (accessed on 24 May 2022) [32]. Multiple sequence alignment was performed using the ClustalX2 server (https://www.ebi.ac.uk/Tools/msa/clustalo/) (accessed on 26 May 2022) [33] and edited with Jalview software [34]. The contiguous open reading frame (ORF) of the DcRyR gene was predicted by ORF Finder (https://www.ncbi.nlm.nih.gov/orffinder/) (accessed on 24 May 2022). Molecular weight and isoelectric point of deduced protein sequences were predicted by ProtParam (http://web.expasy.org/protparam/) (accessed on 24 May 2022). Signal peptide and possible transmembrane (TM) regions were predicted by SignalP v5.0 (http://www.cbs.dtu.dk/services/SignalP) (accessed on 24 May 2022) and TMHMM Server v2.0 (http://www.cbs.dtu.dk/services/TMHMM-2.0) (accessed on 24 May 2022), respectively. The putative domains were predicted using the NCBI conserved domain database [35]. Hydropathy profiles were generated using ExPASy ProtScale (https://web.expasy.org/protscale/) (accessed on 26 May 2022) according to the Kyte–Doolittle method [36], with a window size of 19 amino acids. RyR protein sequences from other species were obtained from GenBank to construct the phylogenetic tree by the maximum likelihood (ML) phylogenetic tree was constructed using RAxML software [37]. Sequences used in this study are shown in Table S4. The phylogenetic tree was visualized and modified by iTOL (https://itol.embl.de/) (accessed on 22 August 2022) [38].

### 2.4. Detection of Alternative Splice (AS) Sites

To detect each putative AS site in each cDNA clone, the assembled full-length of DcRyR was divided into 16 overlapping fragments, and primer pairs were designed with Primer3web (http://bioinfo.ut.ee/primer3/) (accessed on 25 March 2022) [39] to amplify segment gene products using Phanta Max Super-Fidelity DNA Polymerase (Vazyme). Specific diagnostic primers were designed based on the regions flanking the AS sites. Primers used in PCR analysis are listed in Table S2. The PCR system and conditions were the same as those mentioned above. Then, the PCR products were purified and inserted into pJET1.2/blunt Cloning Vector, and 24 positive clones from each sample were sequenced (Sangon Biotech). Finally, the frequencies of each AS variant in samples were recorded.

### 2.5. Detection of RyR Mutations in D. citri Populations

Genomic DNA was extracted from *D. citri* individuals collected in the field from three populations (Table S1) using the Genomic DNA Purification Kit (Thermo Fisher Scientific) following the manufacturer’s instructions. Two pairs of specific primers flanking the M4752 and G4909 sites were used to amplify the 245 bp and 217 bp DNA fragments, respectively (Table S2). PCR was performed in a final volume of 25 mL with 12.5 μL of 2× Taq Master Mix (Vazyme), 1 μL of each primer (10 μM), 1 μL of gDNA, and 9.5 μL of nuclease-free water. The PCR cycle conditions were as follows: initial denaturation of 94 °C for 3 min, 30 cycles of 94 °C for 15 s, annealing at 55 °C for 15 s, 72 °C for 30 s, and a final extension at 72 °C for 7 min. The PCR products were purified using the Gel Extraction Kit (Omega) after 2% agarose gel electrophoresis, and directly sequenced with the forward primer by Sangon Biotech.

### 2.6. Data Analysis of DcRyR Expression

All data were analyzed by one-way analysis of variance followed by the Tukey–Kramer post-hoc test using GraphPad Prism software (GraphPad Software Inc., San Diego, USA). Data are presented as mean ± standard deviation (SD).

## 3. Results

### 3.1. Isolation of DcRyR

**Figure 1:**
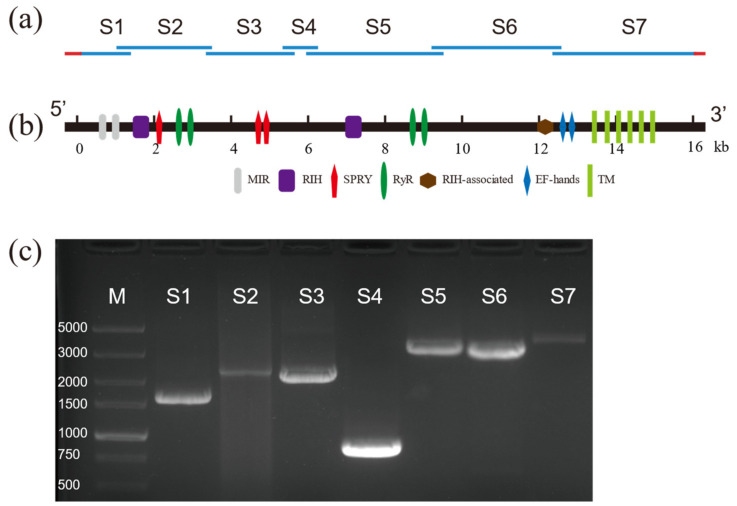
Schematic diagram of cloning strategy and gel electrophoresis of the full-length and alternative splicing of the *Diaphorina citri* ryanodine receptor (DcRyR) cDNA sequence. (**a**) Blue lines indicate the full-length DcRyR open reading frame. Red lines show 5′-UTR and 3′-UTR. S1–S7 represent nucleotide sequences of overlapping cDNA clones. (**b**) Analysis of the DcRyR structure. (**c**) Gel electrophoresis of seven PCR fragments (S1–S7). The PCR products of DcRyR were detected on 2% agarose gel. M indicates DNA marker DL5000 (TaKaRa).

Based on the partial sequence of RyR reported in GenBank, nine pairs of specific primers were used to amplify nine overlapping cDNA fragments from the cDNA of *D. citri* (Figure 1a,c). The overlapping fragments were assembled into a 16,407 bp contiguous sequence containing a 783 bp 5′-untranslated region (UTR), a 232 bp 3′-UTR, and a 15,393 bp ORF (GenBank Accession Number: ON932795). The encoded 5130 amino acid residues predict a protein with a calculated molecular weight of 580.830 kDa and an isoelectric point of 5.41 (Table S3).

### 3.2. Conserved Domain and Phylogenetic Analysis of DcRyR

Analysis of the amino acid sequence of DcRyR indicated that seven conserved domains were present (Figure 1b). The N-terminal region contains an MIR (mannosyltransferase, IP3R, and RyR; Pfam02815) domain (residues 211–392), two RIH (RyR and IP3R homology; Pfam01365) domains (residues 441–640 and 2233–2465), three SPRY (SPla and RyR; cd12877–cd12879) domains (residues 646–797, 1077–1209, and 1539–1690), four RyR (RyR repeated; Pfam02026) domains (residues 852–942, 965–1055, 2830–2920, and 2950–3031), and an RIH-associated (Pfam08454) domain (residues 4004–4121). In addition, six conserved and essential hydrophobic TM segments were predicted in the C-terminal region: TM1 (V4453–L4475), TM2 (Y4656–G4678), TM3 (V4737–L4759), TM4 (F4879–A4901), TM5 (L4927–F4949), and TM6 (I5007–I5026). These regions exhibited high sequence identity between DcRyR and other insect RyRs (Figure S2). The hydropathy profile analysis revealed that these six TM domains corresponded to six highly hydrophobic regions (Figure S3). A glutamate residue that has been proposed to be involved in Ca^2+^ sensitivity in rabbit RyR1 (E^4032^) [40] and RyR3 (E^3885^) [41] was detected in DcRyR (E^4160^). One EF-hand domain pair was also present within the C-terminal region (residues 4201–4228 and 4236–4263), which is a Ca^2+^-binding site originally reported in lobster RyR [42]. Moreover, the highly conserved sequence motif of the pore-forming segment (GXRXGGGXGD) was identified between TM5 and TM6 (residues 4978–4988), which is essential for the ion channel in RyR [43]. Besides, the residues corresponding to I^4897^, R^4913^, and D^4917^ of rabbit RyR1, which play an important role in the activity and conductance of the Ca^2+^ release channel [44], were conserved in DcRyR (I^4986^, R^5002^, and D^5006^).

Multiple sequence alignments between DcRyR protein and its orthologs indicate that they share high similarity (Figure S2). Moreover, RyR identity and similarity between *D. citri* and other species revealed that DcRyR had the highest identity and similarity to RyRs from Hemiptera, being most similar to *Bemisia tabaci* (BtRyR, Hemiptera Aleyrodidae) and *Nilaparvata lugens* (NlRyR, Hemiptera Delphacidae). Notably, the similarity between DcRyR and TcRyR (*Tribolium castaneum*) was 92.28%. The identity of DcRyR with RyRs from *Bombyx mori*, *Acromyrmex echinatior*, and *Drosophila melanogaster* was 79.63%, 77.38%, and 76.57%, respectively. However, the identity of DcRyR in relation to *Homo sapiens* RyR1–3 was only 43–46% (Table S5).

**Figure 2:**
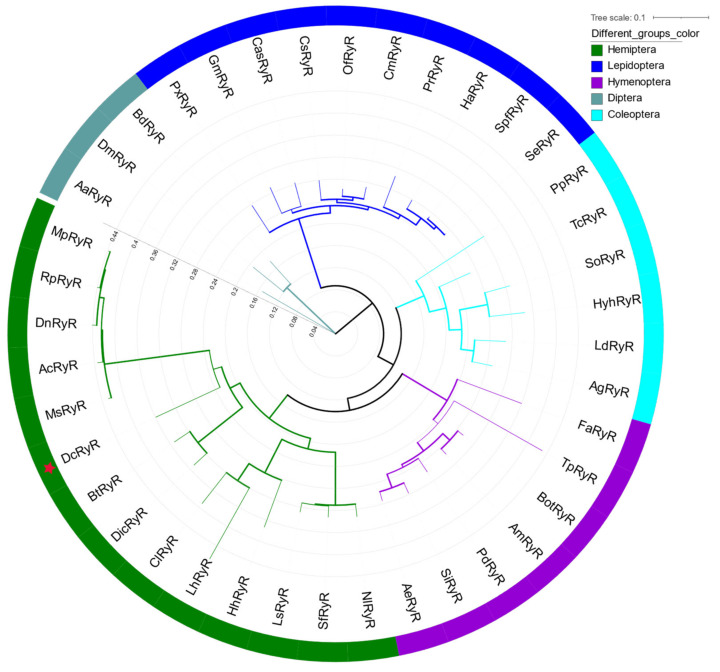
Phylogenetic analysis of *Diaphorina citri* and other selected RyRs. The maximum likelihood tree was constructed using RAxML software. The branch line width represents the level of bootstrap support for each branch. DcRyR is marked with bold text and red asterisk. The accession numbers of sequences used in this tree are given in Table S4.

A phylogenetic tree of RyRs from insects was constructed to evaluate evolutionary relationships. The phylogenetic analysis showed that DcRyR clustered with *D. citri* and *B. tabaci*, and all RyRs of hemipteran insects formed a primary branch (Figure 2).

### 3.3. Detection of AS Sites of DcRyR

**Figure 3:**
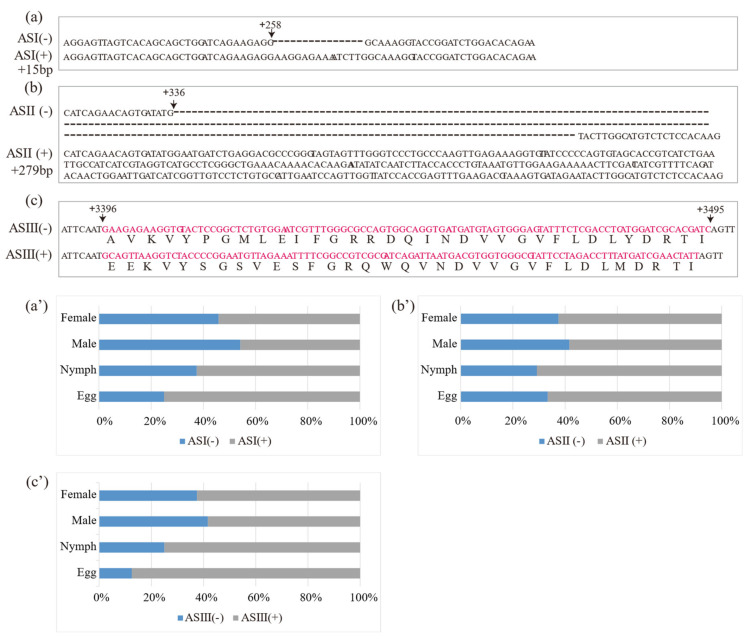
Location of alternatively spliced regions (**a**–**c**) and relative frequencies of alternatively spliced DcRyR (**a′**–**c′**) at various developmental stages (egg, nymph, and adult male and female). The cDNA sequences of ASI (−), ASII (−), and ASIII (−) were aligned with ASI (+), ASII (+), and ASIII (+), respectively.

Three putative AS variants in DcRyR were revealed by the alignment of multiple cDNA clones, and the sequences of PCR products (termed ASI–ASIII) and AS variants with and without AS were named AS (+) and AS (−) (Figure 3a–c), respectively. For ASI and ASII, the generated inclusion and exclusion segments of 15 bp (ASI) and 279 bp (ASII) were located at positions +258 and +336, respectively. ASIII was located between nucleotides 3396 and 3495, constituting a pair of mutually exclusive exons. Diagnostic PCR was used to determine the splicing frequency of the four developmental stage samples (egg, nymph, and adult male and female). Data were collected from 24 positive clones for each sample. All AS (−) were dominant in most developmental stages and parts of the body compared with AS (+), except ASI (+) in male (54%) (Figure 3a′–c′).

### 3.4. DcRyR Expression Profiles

**Figure 4:**
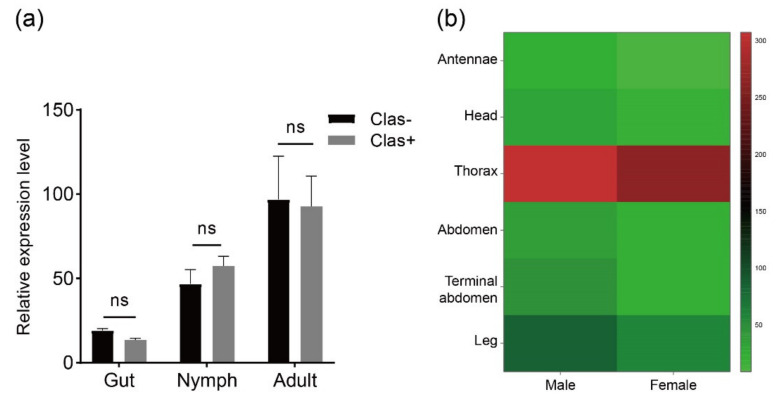
DcRyR expression levels based on RPKM values. (**a**) CLas-free (CLas−) and CLas-infected (CLas+) *Diaphorina citri* in the gut of nymphs and adults. (**b**) Expression levels in different tissues from CLas-free *D. citri* adult (antennae, head, thorax, abdomen, terminal abdomen, and leg).

Transcriptome analysis was performed to investigate the RyR gene expression pattern in tissues at different developmental stages of *D. citri* (Figure 4). Data were obtained from published transcriptome data [45]. DcRyR expression profiles revealed transcript abundance in all developmental stages and tissues tested, and DcRyR exhibited gene-differential and stage-specific patterns. DcRyR expression levels were significantly higher in adults than in nymphs (CLas-, adults vs. nymphs, F = 8.866, *p* = 0.0133; CLas+, adults vs. nymphs, F = 9.636, *p* = 0.0321). Gene expression was also higher in the thorax than in the legs and abdomen (Figure 4b). In addition, RyR gene expression was examined with CLas-free (CLas−) and CLas-infected (CLas+) *D. citri* in the gut of nymphs and adults. The expression levels were not significantly different (Figure 4a).

### 3.5. Detection of DcRyR Mutations

The frequency of two mutations (M4752 and G4909) was studied in field populations of *D. citri* (40 samples) with specific primers. None of the samples had mutations at these sites.

## 4. Discussion

Diamide insecticides are a relatively new type of insecticide with strong insecticidal action and low toxicity to mammals [46], and RyR molecular information is significant to their development to realize their immense potential in insect control. Since 2007, these insecticides have been extensively used on numerous crop species all over the world [47]. However, full-length RyR cDNA isolation is imperative to fully understand the role of RyRs. Since the isolation of the first insect RyR from *D. melanogaster* in 1994, full-length RyRs have been identified in several insect species, including lepidopteran species such as *Plutella xylostella* (Plutellidae) [48,49], coleopteran species such as *T. castaneum* (Tenebrionidae) [50], hymenopteran species such as *Encarsia formosa* (Aphididae) [51], hemipteran species such as *Sogatella furcifera* (Delphacidae) [52], and dipteran species such as *Bactrocera dorsalis* (Tephritidae) [53]. However, there is no detailed report on RyR in *D. citri*. In the present study, full-length RyR cDNA was isolated and identified from *D. citri*, which is the most important pest of citrus worldwide because it transmits the pathogen that causes HLB, a devastating citrus disease.

### 4.1. Isolated and Characterization of DcRyR

The Ca^2+^ channel protein RyR, which is essential and conserved, has similar types, numbers, and positions of conserved domains in different organisms. Multiple alignment of the deduced amino acid sequence of DcRyR revealed high identities (between 77% and 84%) with other known insect RyRs, but low identities (between 43% and 56%) with nematode and mammalian RyRs (Table S5). Analysis of the DcRyR amino acid sequence revealed that the conserved structural domains of DcRyR included three MIR domains, two RIH domains, four RyR domains, one RIH-associated domain, and three SPRY domains (Figure 1b), which were also present in RyRs of *P. xylostella* [48,49], *N. lugens* [54], *B. dorsalis* [53], and *Leptinotarsa decemlineata* (Chrysomelidae) [55]. The phylogenetic analysis also demonstrated the conservation of insect RyRs. While other principal branches in the tree were established based on insect order, the DcRyR and RyR branches of the other hemipteran insects formed a branch (Figure 2), indicating that RyR genes may have evolved after the diversification of insect order. RyR has been investigated as a molecular target for the creation of pesticides because of its significance and conservation in insects [56,57].

### 4.2. AS Site of DcRyR

Alternative splicing is an important mechanism in the regulation of transcription [58]. AS variants have been observed in several insects, such as *P. xylostella* (Plutellidae) [48,49], *Heliothis virescens* (Noctuidae) [59], *Cnaphalocrocis medinalis* (Pyralidae) [60], *Helicoverpa armigera* (Noctuidae) [61], *Ostrinia furnacalis* (Pyralidae) [62], *N. lugens* (Delphacidae) [54], *Toxoptera citricida* (Aphididae) [63], *E. formosa* (Aphididae) [51], and *D. melanogaster* (Drosophilidae) [64]. AS RyR genes have been found in DcRyR as well. In the present study, three AS sites (ASI–ASIII) were found in DcRyR, whereas all AS sites were excluded in conserved domains (Figure 4). A mutually exclusive AS of RyR in the SPRY domain could be associated with the immune response [65]. However, the functions of AS variants in DcRyR should be further examined.

### 4.3. DcRyR Expression Profiles

RyR expression profiles in different tissues may be closely related to their function. RyRs play an important role in the regulation of calcium homeostasis, in which Ca^2+^ is one of the secondary messengers and participates in numerous biological processes including nerve conduction and muscle contraction [66]. Thus, RyR was highly expressed in tissues in which the muscular system was widely distributed. Among the body sections, the thorax had higher levels of DcRyR expression than other tissues (Figure 3), which may be compatible with the functions of the Ca^2+^ channel. *P. xylostella* (Plutellidae), *B. dorsalis* (Tephritidae), *L. decemlineata* (Chrysomelidae), *S. furcifera* (Delphacidae), and *Pieris rapae* (Pieridae) showed similar outcomes, with more expression in the thorax than in the abdomen of adults [52,53,54,55,67,68].

### 4.4. Detection of DcRyR Mutations

Diamide resistance-related amino acid residue mutations I4790M/K and G4946E were found in RyR from *P. xylostella* [69,70,71]. Similar mutations have been reported in *Tuta absoluta* [72], *Chilo suppressalis* [73,74], *Adoxophyes honmai* [75], and *Spodoptera exigua* [76,77]. Mutations in DcRyR were located at M4752 and G4909. However, these mutations were not detected in all samples, which might be because a sensitive *D. citri* strain was used in the study.

## 5. Conclusions

In the present study, the ryanodine receptor gene in *D. citri* was identified and cloned, and its gene structure was analyzed. The deduced 5130 amino acid residues of DcRyR showed a reasonable degree of identity with known RyRs along the whole molecule and shared several common structural features. The expression level at different developmental stages and tissues from *D. citri* was also confirmed, the results showing that the mRNA expression level in adults was significantly higher than in other developmental stages, and the thorax exhibited the highest expression level compared with the legs and abdomen. Moreover, we reported three AS variants in DcRyR gene, and the frequencies of AS variants were investigated at each developmental stage of *D. citri*. In addition, diamide resistance-related amino acid residue mutations I4790M/K and G4946E were not found in DcRyR. These findings can provide fundamental knowledge for comprehending the underlying molecular mechanisms of diamide pesticide resistance.

## Data Availability

All the data generated in this work was provided in the article.

## Supplementary Materials

The following supporting information can be downloaded at: https://www.mdpi.com/article/10.3390/life12122005/s1, Figure S1: Schematic representation of RyR sequence comparison, the accession numbers of sequences used in this tree are given in Table S4; Figure S2: Amino acid sequence alignment of transmembrane domains at the C-terminus of DcRyR and other species RyR isoforms, the abbreviations are described in Table S4; Figure S3: Hydropathy profile of DcRyR C-terminus amino acids; Table S1: The detailed information of collected samples; Table S2: Primers used in the experiment; Table S3: Characterization of the ryanodine receptor gene from *Diaphorina citri*; Table S4: The accession numbers of the sequences used in this study; Table S5: Sequence identity (I) and similarity (S) between *Diaphorina citri* and other species RyR.
