# Supplementary material for: Functional Characterization of the Ryanodine Receptor Gene in Diaphorina citri
Source: Life (Basel). 2022 Dec 1;12(12):2005. doi: 10.3390/life12122005 (PMC9785964; doi:10.3390/life12122005)
Supplement: Supplementary file 1 [file life-12-02005-s001.zip › Tables S1-S5.pdf]

Table S1 The detailed information of collected samples

Population	Sample size	Latitude	Longitude	Altitude
1	8	23°9’15’’	113°21’13’’	40.2m
2	14	23°40’49’’	114°15’39’’	72.8
3	18	23°9’14’’	113°22’0’’	50.5m

Table S2 Primers used in the experiment

Product name	Primer sequences (5’-3’)	Length (bp)	Use of primers
S1	F: CGCGAGTTATTTCTCCCCGT	1621	Full length cloning
	R: GTCTTCTATTCACCTGCAGAGC		
S2	F: GTCAGGAGAAGGATGACCAGAA	2331	
	R: ACCATTTGCCGCTAGACACG		
S3	F: GTGAGACGGTAAGAACATTGC	2191	
	R: CACCGGTCTTCCTCTGGGAT		
S4	F: GTGAGACGGTAAGAACATTGC	856	
	R: CACCGGTCTTCCTCTGGGAT		
S5	F: ATGACGGATATTGCACCGGACAGCATTGAAAACATCCGCA	3365	
	R: TCCCGATCCTTCTTCTTCTCCT		
S6	GGATGGAATATTGAACACAGCGAGGCAGATGTGCCT	3183	
	AACGCTGCAAGAGTTCATAAGAC		
S7	F: TGGTGATGAAAACTCTACAGCTGGGTATTGCTGTGCTACG	3846	
	R: CGGATTTACAGTCTCACAGTTG		
5’-UTR	F: CCAGATTCGCAGAGAACTTAG	961	
	R: GATAGGTCAGGAGGGATGTTCTTGTCAGCAATATTCTCGA		
3’-UTR	F: CATCTGTGGTATAGGCAAGGAC	482	
	R: CGGATTTACAGTCTCACAGTTG		
ASI	F: CCAGGTAGAAAATGATTCACTGAC	292	Alternative splice
	R: AGTGCCGATTACCGAAACCT		
ASII	F: CCAGGTAGAAAATGATTCACTGAC	399	
	R: TGTGACTAACTCCTGGAGTGC		
ASIII	F: CCTGACCTATCCCAATGTGTG	168	
	R: CATATCACTGTTCTGATGGCG		
ASIV	F: CTGGACACAGAACACTTCTC	161	
	R: TACGGTCCACCAACACGCTTC		
ASV	F: CGAGCAGAGAAGAATTATGCC	415	
	R: AATACTTGAGCTGGTTCACATCCT		
M4752	F: CGATATATCCGGTCTAGGCAG	245	Sanger sequencing
	R: CGTTATTGATCGACGTGGATCG		
G4909	F: CGTTCTTGTACAGTCTATGGTATTTC	217	
	R: TAACGTCAGTGTACAGCTGC		

Note: F, forward primer; R, reverse primer.

Table S3 Characterization of the Ryanodine receptor gene from *Diaphorina citri*

Sequence ID	Number of Amino Acid	Molecular Weight	Theoretical pI	Instability Index	Aliphatic Index	Grand Average of Hydropathicity
DcRyR	5130	580830.34	5.41	46.18	84.36	-0.335

Table S4 The accession numbers of the sequences used in this study

Name	Accession no.	Species
SifRyR	XP_025425821.1	*Sipha_flava*
MsRyR	XP_025202171.1	*Melanaphis sacchari*
HhRyR	XP_024217614.1	*Halyomorpha halys*
ClRyR	XP_024086009.1	*Cimex lectularius*
ApRyR	XP_016661950.1	*Acyrthosiphon pisum*
DnRyR	XP_015367530.1	*Diuraphis noxia*
BrmRyR	XP_001900491.1	*Brugia malayi*
AaRyR	XP_001657320.2	*Aedes aegypti*
MmRyR1	NP_033135.2	*Mus musculus*
MmRyR2	NP_076357.2	*Mus musculus*
MmRyR3	NP_808320.2	*Mus musculus*
DrRyR1b	NP_001096041.1	*Danio rerio*
OcRyR1	NP_001095188.1	*Oryctolagus cuniculus*
OcRyR2	NP_001076226.1	*Oryctolagus cuniculus*
OcRyR3	NP_001076231.1	*Oryctolagus cuniculus*
HsRyR1	NP_000531.2	*Homo sapiens*
HsRyR2	NP_001026.2	*Homo sapiens*
HsRyR3	NP_001027.3	*Homo sapiens*
LhRyR	JAQ15715.1	*Lygus hesperus*
BmRyR	XP_012544769	*Bombyx mori*
SrRyR1	CEF62113.1	*Strongyloides ratti*
TtRyR	CDW52896.1	*Trichuris trichiura*
DmRyR	BAA41470.1	*Drosophila melanogaster*
CeRyR	BAA08309.1	*Caenorhabditis elegans*
LcRyRbeta	BAA04647.2	*Lithobates catesbeianus*
LcRyRalpha	BAA04646.1	*Lithobates catesbeianus*
AcRyR	AKM95171.1	*Aphis citricidus*
DicRyR	AKM95170.1	*Dialeurodes citri*
MpRyR	AJA41114.1	*Myzus persicae*
TcRyR	AIU40166.1	*Tribolium castaneum*
GmRyR	AIP90097.1	*Grapholitha molesta*
LsRyR	AIA23858.1	*Laodelphax striatellus*
NlRyR	AIA23857.1	*Nilaparvata lugens*
CsRyR	AIA23856.1	*Chilo suppressalis*
HaRyR	AIA23855.1	*Helicoverpa armigera*
BdRyR	AHY02115.1	*Bactrocera dorsalis*
LdRyR	AHW99830.1	*Leptinotarsa decemlineata*
SfRyR	AHW99829.1	*Sogatella furcifera*
CasRyR	AHN16453.1	*Carposina sasakii*
PrRyR	AGI62938.1	*Pieris rapae*
OfRyR	AGH68757.1	*Ostrinia furnacalis*

BtRyR	AFK84957.1	*Bemisia tabaci*
CmRyR	AFI80904.1	*Cnaphalocrocis medinalis*
SeRyR	AFC36359.1	*Spodoptera exigua*
PxRyR	AET09964.1	*Plutella xylostella*
AmRyR	XP_006569098.1	*Apis mellifera*
BotRyR	XP_048270099.1	*Bombus terrestris*
SiRyR	XP_039305071.1	*Solenopsis invicta*
PdRyR	XP_015173032.1	*Polistes dominula*
FaRyR	XP_011300758.1	*Fopius arisanus*
AeRyR	XP_011054963.1	*Acromyrmex echinatior*
TpRyR	XP_023317849.1	*Trichogramma pretiosum*
HyhRyR	QEE14187.1	*Hypothenemus hampei*
PpRyR	XP_031356017.1	*Photinus pyralis*
SoRyR	XP_030749978.1	*Sitophilus oryzae*
AgRyR	XP_018566920.1	*Anoplophora glabripennis*

Table S5 Sequence identity (I) and similarity (S) between *Diaphorina citri* and other species ryanodine receptor

I<br>S	DcRyR	BtRyR	NlRyR	AeRyR	DmRyR	CeRyR	TcRyR	BmRyR	PxRyR	DrRyR1b	OcRyR1	OcRyR2	OcRyR3	MmRyR1	MmRyR2	MmRyR3	HsRyR1	HsRyR2	HsRyR3
DcRyR		**83.59**	**83.26**	**77.38**	**76.57**	**44.97**	**80.97**	**79.63**	**78.34**	**42.86**	**43.25**	**45.62**	**43.81**	**43.45**	**45.51**	**43.65**	**43.14**	**45.60**	**43.94**
BtRyR	**93.69**		83.35	76.95	75.76	45.03	80.60	78.96	77.76	42.63	43.20	45.71	43.74	43.37	45.59	43.78	43.08	45.73	43.94
NlRyR	**92.73**	93.41		78.15	76.62	44.61	80.99	79.38	78.40	42.80	43.01	45.30	43.57	43.10	45.23	43.47	42.75	45.32	43.70
AeRyR	**90.03**	89.74	89.85		80.86	44.31	78.27	78.11	76.87	42.77	43.12	45.38	43.74	43.36	45.35	43.63	43.03	45.40	43.91
DmRyR	**89.74**	89.04	89.03	92.32		44.33	77.54	77.66	76.40	41.70	42.50	44.43	42.94	42.64	44.40	42.80	42.38	44.39	43.03
CeRyR	**73.47**	73.25	72.86	72.87	72.48		45.69	45.16	44.56	36.83	37.66	39.64	37.88	37.71	39.59	37.89	37.53	39.75	38.23
TcRyR	**92.28**	91.86	91.51	90.61	90.21	74.11		81.56	79.96	42.74	43.096	45.33	44.06	43.30	45.17	43.82	42.87	45.33	44.05
BmRyR	**91.19**	90.81	90.09	90.01	89.71	73.81	91.64		89.96	43.04	43.554	45.55	43.99	43.71	45.46	43.79	43.43	45.57	44.10
PxRyR	**90.08**	89.93	89.45	89.19	88.83	72.93	90.51	95.35		42.19	42.906	44.99	43.29	42.98	44.90	42.94	42.82	45.01	43.28
DrRyR1b	**70.83**	71.02	70.92	71.00	70.54	66.60	70.95	71.23	70.3		75.443	64.51	64.32	75.19	64.29	64.13	74.98	64.51	64.77
OcRyR1	**70.67**	70.79	70.63	70.95	70.42	66.52	71.15	70.98	70	88.69		64.40	64.18	95.86	64.22	63.90	96.29	64.46	64.39
OcRyR2	**72.40**	72.75	72.04	72.23	71.88	68.71	72.69	72.76	72.19	85.76	85.06		67.12	64.37	97.062	66.95	64.15	98.67	67.41
OcRyR3	**70.94**	70.90	70.47	70.54	70.29	67.45	71.37	71.11	70.51	84.49	84.34	86.81		64.19	66.91	94.74	63.93	67.08	96.35
MmRyR1	**70.70**	70.81	70.49	70.84	70.46	66.52	70.95	70.86	69.91	88.87	98.47	85.37	84.42		64.18	63.96	95.38	64.43	64.42
MmRyR2	**72.55**	72.72	72.06	72.28	71.85	68.73	72.77	72.63	72.06	85.60	84.98	99.20	86.73	85.19		66.90	64.01	97.20	67.31
MmRyR3	**70.52**	70.67	70.33	70.43	70.07	67.29	71.22	70.96	70.09	84.55	84.13	86.68	97.97	84.14	86.58		63.75	66.97	95.56
HsRyR1	**70.89**	70.80	70.62	71.09	70.53	66.55	71.06	71.13	70.03	88.94	98.53	85.13	84.16	98.42	84.99	83.98		64.19	64.18
HsRyR2	**72.51**	72.85	72.14	72.39	71.94	68.73	72.86	72.89	72.36	85.76	85.16	99.72	86.87	85.41	99.32	86.74	85.21		67.35
HsRyR3	**70.93**	70.99	70.45	70.71	70.31	67.69	71.48	71.26	70.40	84.77	84.59	86.99	98.71	84.56	86.97	98.53	84.36	87.07	

The percentage of similarity (lower left triangle) and identity (upper right triangle) are illustrated. Abbreviations and GenBank entries for the RyR isoforms are described in Table S4.
