# Supplementary figures and images for: Functional Characterization of the Ryanodine Receptor Gene in Diaphorina citri
Source: Life (Basel). 2022 Dec 1;12(12):2005. doi: 10.3390/life12122005 (PMC9785964; doi:10.3390/life12122005)

Figure S1

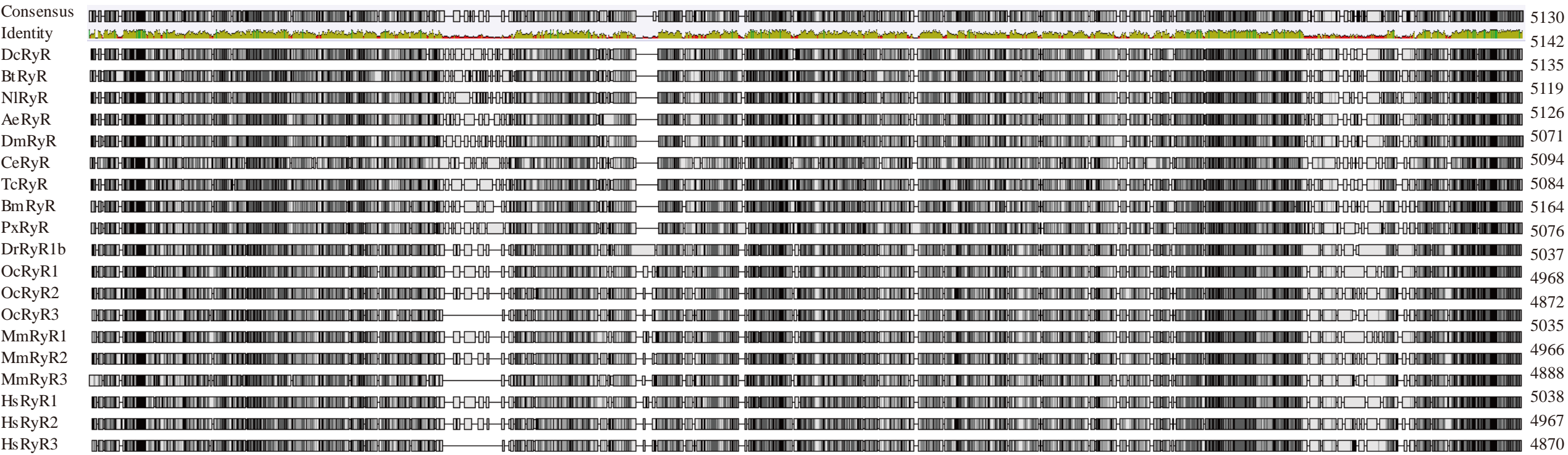

Figure S2

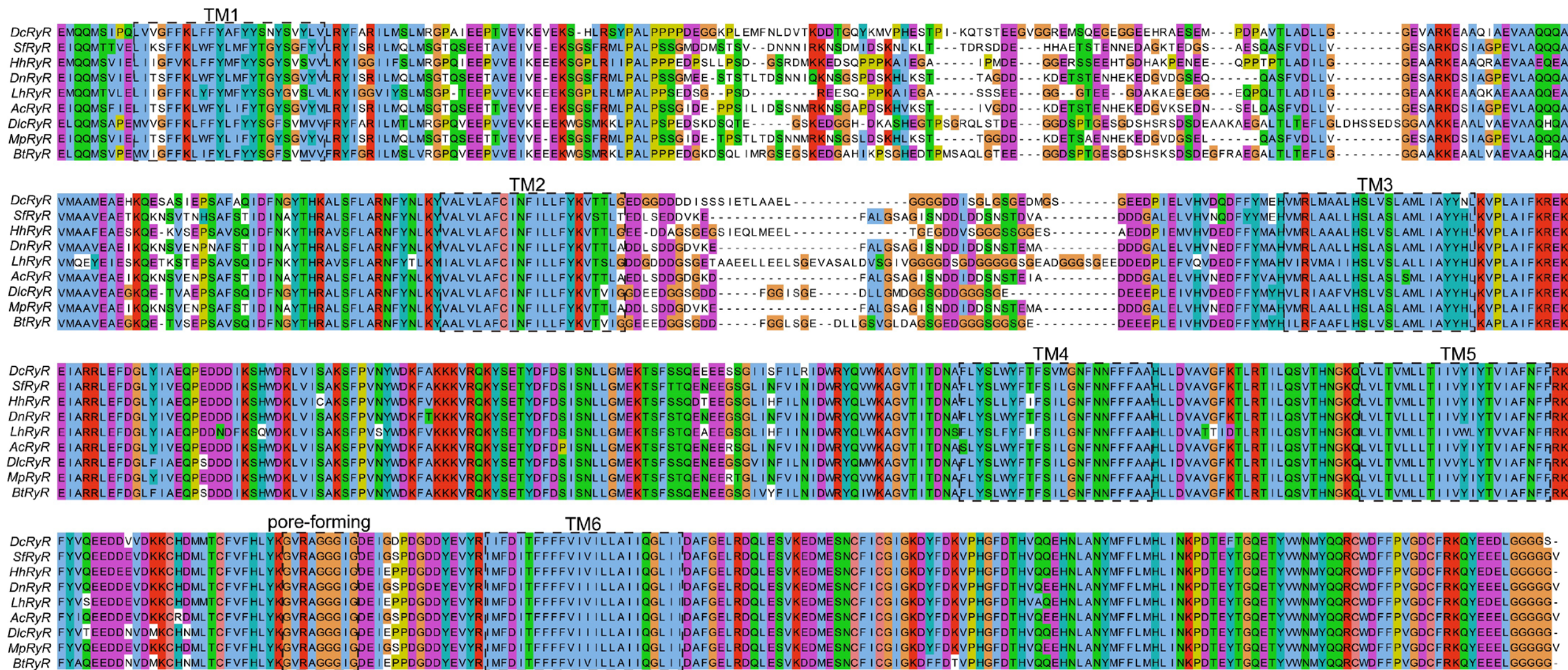

Figure S3

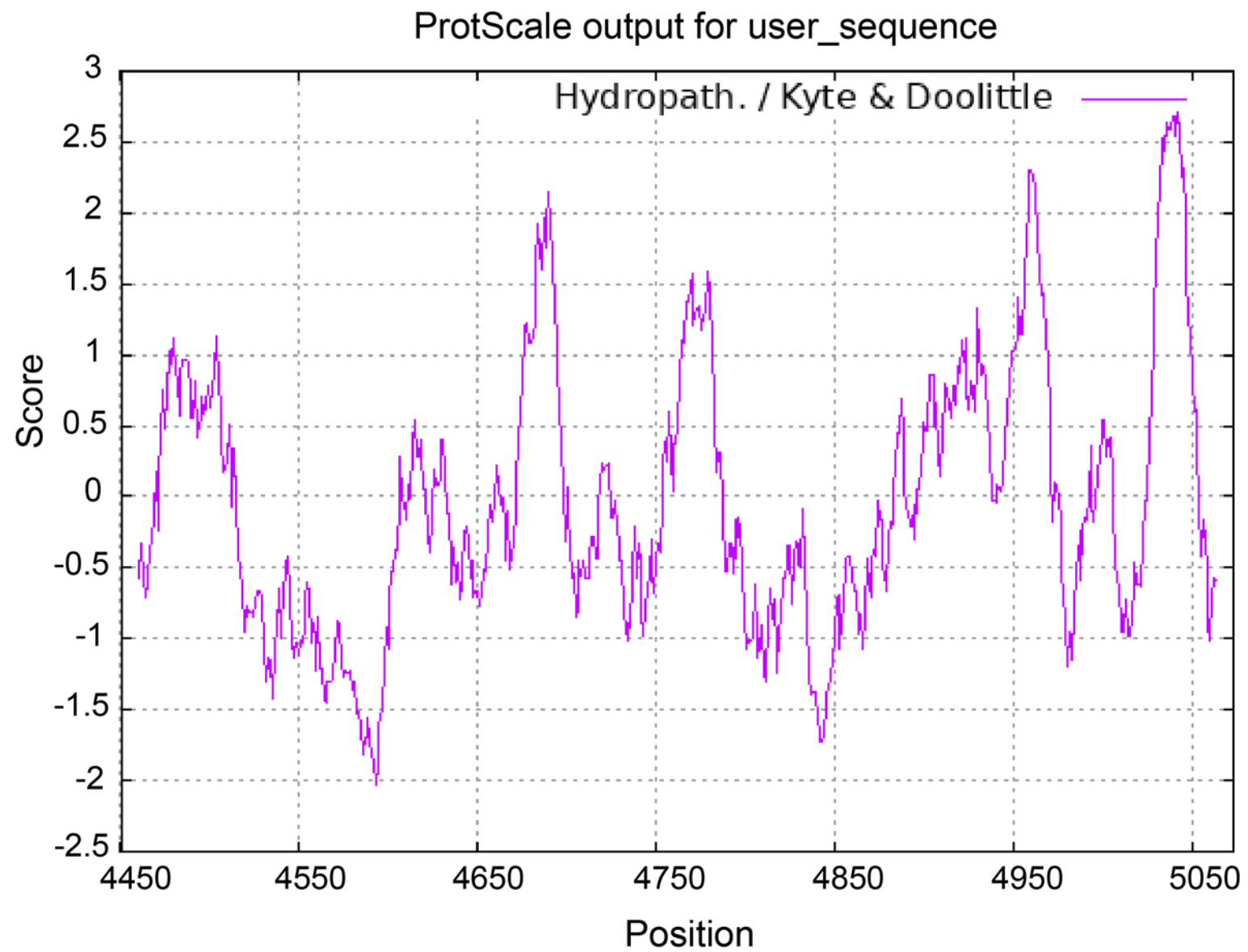

Supplement: Supplementary file 1 [file life-12-02005-s001.zip › Figures S1-S3.pdf]
